# FTO haplotyping underlines high obesity risk for European populations

**DOI:** 10.1186/s12920-019-0491-x

**Published:** 2019-03-13

**Authors:** Vladimir Babenko, Roman Babenko, Junaid Gamieldien, Arcady Markel

**Affiliations:** 1 Federal Research Center Institute of Cytology and Genetics SB RAS, 10 Lavrentieva Ave, Novosibirsk, Russian Federation 630090; 2 Novosibirsk State University, 2 Pirogova Str, Novosibirsk, Russian Federation 630090; 3 South African National Bioinformatics Institute/SAMRC Unit for Bioinformatics Capacity Development, University of the Western Cape, Cape Town, 7535 South Africa

**Keywords:** Genomics, Population genetics, Obesity, Haplotypes, *FTO* gene

## Abstract

**Background:**

Fat mass and obesity-associated (*FTO)* gene has been under close investigation since the discovery of its high impact on the obesity status in 2007 by a range of publications. Recent report on its implication in adipocytes underscored its molecular and functional mechanics in pathology. Still, the population specific features of the locus structure have not been approached in detail.

**Methods:**

We analyzed the population specific haplotype profiles of *FTO* genomic locus identified by Genome Wide Association Studies (GWAS) for the high obesity risk by examining eighteen 1000G populations from 4 continental groups. The GWAS SNPs cluster is located in the *FTO* gene intron 1 spanning around 70 kb.

**Results:**

We reconstructed the ancestral state of the locus, which comprised low-risk major allele found in all populations, and two minor risk-associated alleles, each one specific for African and European populations, correspondingly. The locus structure and its allele frequency distribution underscore the high risk allele frequency specifically for the European population. South Asian populations have the second highest frequency of risk alleles, while East Asian populations have the lowest. African population-specific minor allele was only partially risk-associated. All of the GWAS SNPs considered are manifested by low risk alleles as reference (major) ones (*p* > 0.5) in each of the continental groups. Strikingly, rs1421085, recently reported as a causal SNP, was found to be monomorphic in ancestral (African) populations, implying possible selection sweep in the course of its rapid fixation, as reported previously.

**Conclusion:**

The observations underscore varying *FTO* -linked risk in the manifestation of population specific epidemiology of genetically bound obesity. The results imply that the *FTO* locus is one of the major genetic determinants for obesity risk from GWAS SNPs set.

**Electronic supplementary material:**

The online version of this article (10.1186/s12920-019-0491-x) contains supplementary material, which is available to authorized users.

## Introduction

Assessment of genetic risk to being overweight is the high priority task actively pursued in studies due to a wide spread epidemiological problem of obesity in many countries. The series of works on genetic risk assessment by GWAS published in 2007 [1–4] underscored *FTO* as the major obesity risk locus associated with up to *P* < 2E^− 223^ with Body Mass Index (BMI) [5].

The *FTO* gene is expressed in a broad range of tissues since it belongs to housekeeping genes class and maintains CpG islands at promoters. It spans more than 410 kb, which is atypically large for a housekeeping gene. It encodes a 2-oxoglutarate-dependent oxygenase, which performs oxidative demethylation of RNA/DNA. *FTO* and surrounding genes are highly conserved across mammalian species. In particular, it is enriched with ultra conserved non-coding elements (UCNE): 10 UCNEs reside within the gene (20 fold enrichment), while only around 4000 UCNEs are observed genome wide [6, 7].

Upon initial GWAS identification of *FTO* intron 1 as a highly obesity-associated locus in 2007 by 4 independent teams [1–4], the subsequent analysis of *FTO* molecular mechanics implicating it as such a profound association marker immediately emerged in the same year [8]. It pointed that *FTO* pays a role in the hypothalamus arcuate nuclei where it mediates energy balance and feeding behavior. Subsequent studies also mostly implicated the hypothalamus as an etiological source of the obesity manifestation, involving such genes as *MC4R*, *MC3R*, *SLC6A14*, *TMEM18*, *POMC*, *BDNF*, *NEGR1* [9].

While a set of long range enhancers at *FTO* has been corresponded previously [6], recent publication by Claussnitzer et al. [10] reported that *FTO* affects the expression of neighboring *IRX3/IRX5* genes specifically in adipocytes. It was experimentally confirmed that the *FTO* intron 1 high risk locus is involved in superenhancer activation [10] and regulates the expression of flanking *IRX3* and *IRX5* loci, which is vital for the maturation mode of adipocytes that is mediated by specific chromatin conformation profile. The rs1421085 alternative allele disrupts the transcription factor binding site (TFBS) for the ARID5 repressor, which leads to doubling of *IRX3/IRX5* expression resulting in pathologic consequences for adipose tissue [10]. In particular, it leads to decreasing mitochondrial thermogenesis and the increase of lipid storage due to the reduction of the adipocyte browning rate mediated by increased *IRX3* expression. This finding apparently shifts the disease cause from brain to adipose tissue in overweight genetic etiology paradigm, on one side, and on another it positions *FTO* as a ‘drive-through’, nonessential gene for the obesity trait [11].

In spite of convincing results in elucidating casual impact of the GWAS SNP in adipocytes [10], there exists a balancing mechanism, as was reported in lean versus obese children study with the ‘disease-risk’ associated rs1421085 genotype [12]. Also, in the subsequent discussion in New England Journal of Medicine [10], the authors raised the concern that other factors, such as epitranscriptomic [13], or feeding behavior [14, 15], could be involved in *FTO* mediated etiology of obesity, which was met with partial counter-argumentation from the authors of original work [16]. Subsequent studies confirmed that alteration of the *IRX3* gene expression rate is mediated by rs1421085 risk allele [12, 17]. A detailed review on the possible impacts of *FTO* on the obesity trait is presented in [9].

While the mechanism elucidated in [10] implies that all other GWAS SNPs in the region are just invoked due to high linkage disequilibrium in the region, other GWAS assessed SNPs in the region maintain even higher association confidence (Table 1). This might imply a pleiotropic effect of the locus in various tissues. Notably, Bell et al., 2010 [18] identified the spanning UCNE with enhancer histone marks haplotype-specific methylation shifts within the region, characterized by coordinated alteration of CpG content mediated by GWAS SNPs in particular. Similar methylation profile alterations for rs9939609 –bound haplotypes were reported in [19, 20]. Thus, multiple casual aspects could be added to that proposed by [10].

**Table 1:** Fifthteen ordered SNPs elucidated by GWAS in the FTO intron 1 73 kb region

SNP_id	Risk	Alt	GWAS *P*_value	Flanking NTs
rs6499640	A	G	4.00E-13	AGG
rs9940128^a^	A	G	4.00E-23	CGG
rs1421085	C	T	6.00E-39	ATA
rs1558902	A	T	2E-223	GTT
rs1121980	A	G	4.00E-08	TGT
rs62033400	G	A	2.00E-14	AAA
rs17817449^a^	G	T	2.00E-12	CTG
rs8043757	T	A	5E-110	AAC
rs8050136	A	C	2E-58	TCA
rs11075990^a^	G	A	2E-51	CAT
rs9939609	A	T	4E-51	TTG
rs7202116^a^	G	A	2.00E-10	CAT
rs7185735	G	A	1E-79	TAG
rs17817964	T	C	1.00E-10	ACA
rs12149832^a^	A	G	5.00E-22	CGT

Change of CpG dinucleotide content is marked with ‘^a^’. ‘Risk’ – risk-associated allele; ‘Alt’ – reference (major) allele

The current point on *FTO* intron 1 haplotype profiling in continental supergroups is not exhaustively elaborated. In [21] it was stressed that *FTO* intron 1 is a GWAS SNPs cluster comprising of 15 SNPs and a partial linkage analysis suggested significant population specific risk variation. Here, we analysed this 73 kb SNPs cluster by performing haplotype analysis in 18 populations which comprise 4 supergroups from the 1000 Genomes (1000G) project.

## Materials and methods

### GWAS SNPs

**Fig. 1:**
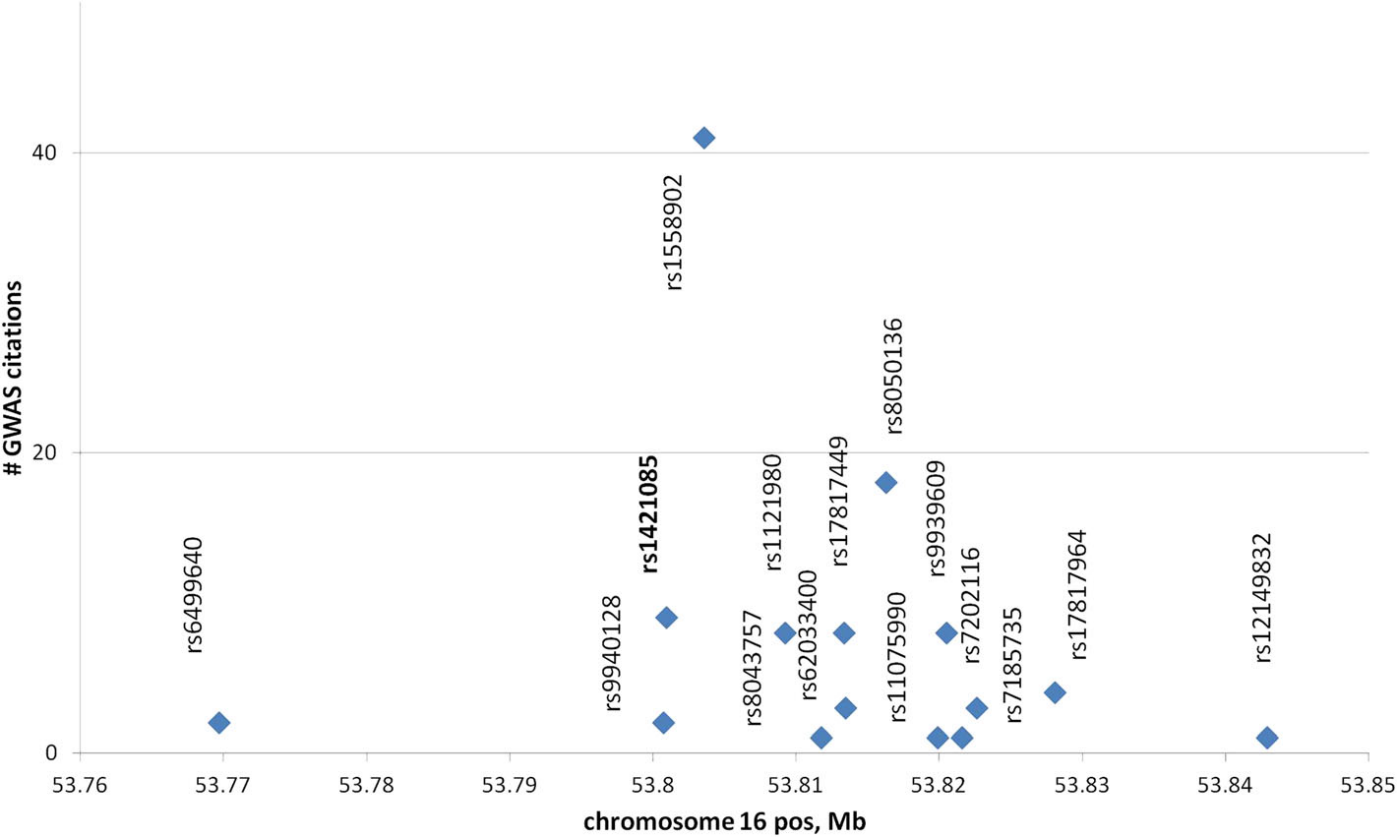
Target cluster of SNPs within *FTO* intron 1 across the chromosome and number of GWAS citations (see Additional File 1, Table S1). Bold typed is a casual SNP elucidated in (Claussnitzer et al., 2015)

We downloaded 14 obesity related SNPs located in *FTO* spanning 42 kb of intron 1, along with their frequencies published in GWAS (Additional file 1: Table S1). We also included a 31 kb-distal GWAS SNP located at the beginning of the cluster (rs64999640; Fig. 1). Overall, 15 target SNPs spanning 73 kb are presented in Table 1, Fig. 1. The risk alleles were unambiguously assigned throughout GWAS reports (Additional file 1: Table S1) for all of continental supergroups (East Asian, African, European, South Asian).

The choice was made according to formal criteria of SNP clustering within a 100 kb region with empirically chosen borderline SNPs GWAS association significance rate of *P* < 5E^− 13^ and overall number > 12.

### 1000 Genomes data

We downloaded a subset of 1000 Genomes phase 3 data (http://www.internationalgenome.org/) [22] for 4 supergroups: 1) African (504 individuals total); 2) East Asian (504 individuals total), 3) European (503 individuals total), 4) South Asian (489 individuals total). We omitted American native supergroup (AMR) from 1000G since it significantly overlaps with other 4 supergroups by allele profiles, for *FTO* locus in particular (1000GPC et al., 2012; personal observation). Overall, 2000 individuals were analyzed. A detailed description of the populations is located in (Additional file 1: Table S2.

### Haplotype analysis

We used the haplotype profiles as provided in 1000 Genomes phase 3 release throughout the study. We inferred the ML haplotype phylogenetic tree for 10 SNPs within the *FTO* locus by DNAML program from phylip package (http://evolution.genetics.washington.edu/phylip.html).

The pairwise comparison of haplotype frequencies was carried out by Conventional F-test implemented in Arlequin software [23]. In particular, average number of pairwise differences between populations *X* and *Y*: *P(X,Y)* was calculated, then average pairwise differences within populations has been carried out denoted by *P(X), P(Y)*. Lastly, the corrected pairwise differences between populations were calculated as

$$ {P}^{\hbox{'}}\left(X,Y\right)=P\left(X,Y\right)-\left(P(X)+P(Y)\right)/2 $$

*P* values were assessed by Monte-Carlo Method based on 100,000 simulations for each pair.

*AMOVA* (Analysis of Molecular Variance; [24]) analysis of variance based on group wise analysis with 18 populations assembled into 4 correspondent groups has been carried out using Arlequin mainframe).

We used XLStat software for Principal Components analysis (www.xlstat.com).

## Results

### Fifteen GWAS SNPs frequency profile in obesity-risk/healthy dichotomy

**Fig. 2:**
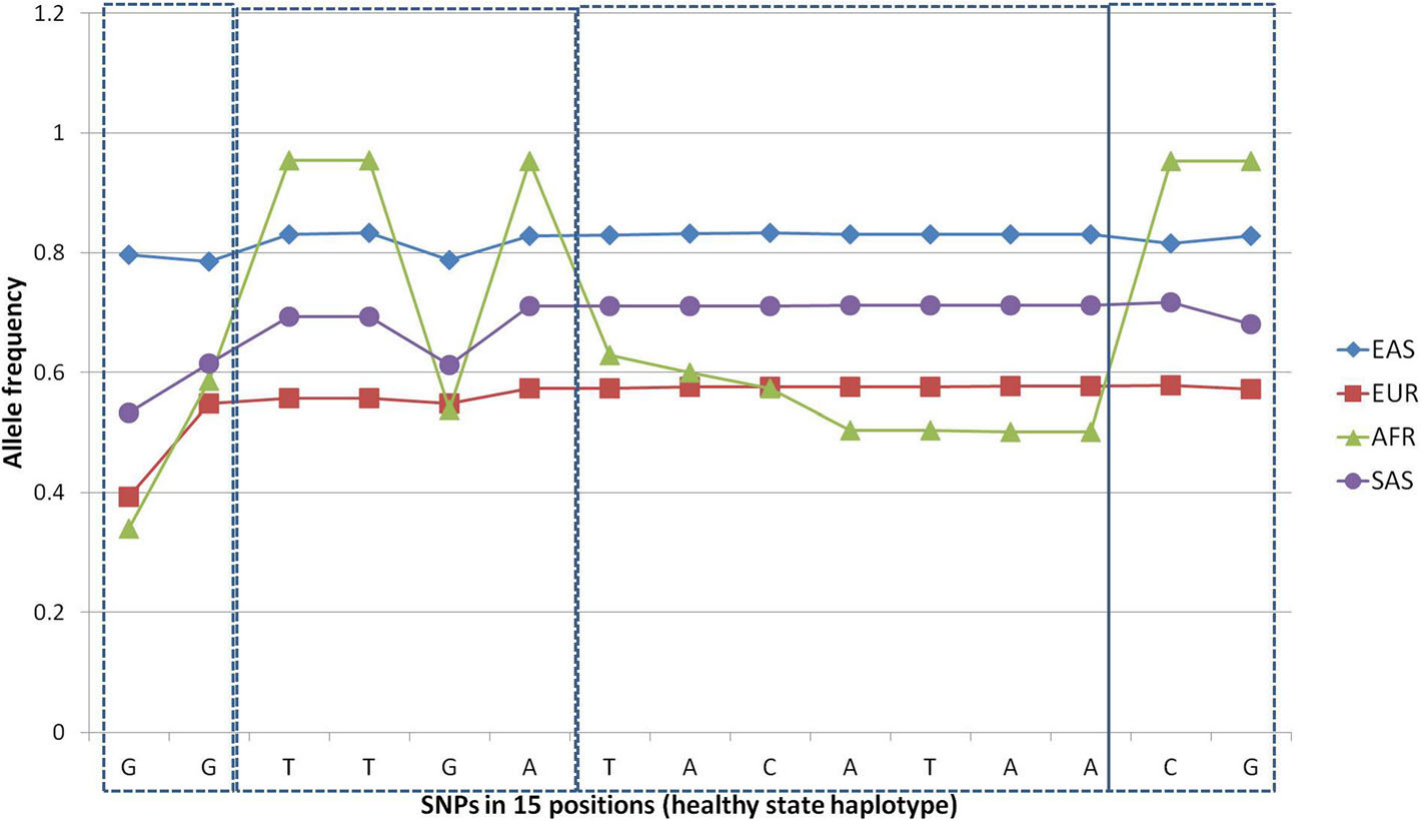
SNPs frequencies distributions (major low-risk haplotype total wise; Fig. 4) and four haplotype blocks in AFR population. Positions correspond to SNPs in Table 1

We present fifteen target GWAS SNPs-specific frequency profiles in Fig. 2. According to allele frequencies distribution and previous haplotype research [21], we segregate the locus into 4 haploblocks (Fig. 2). The haploblocks partition was majorly ‘supervised’ by African populations group due to the allele frequency profiles (Fig. 2) and, to a minor extent, other (non-European) populations. Note that positions 3 (rs1421085), 4 (rs1558902), 6 (rs62033400), 14 (rs17817964), 15 (rs12149832) are highly monomorphic in the ancestral population (AFR), implying that they can shift the original (ancestral) obesity status during the course of evolution. In particular, no drastic adipocyte specific pathophysiology of obesity due to the rs1421085 alternative allele [10] should be observed in African population. Another clear observation from Fig. 2 is that the European population shares the smallest ratio of low-risk alleles across all polymorphisms considered. Notably, all 15 SNPs are linked in EAS populations by *r*^*2*^ statistic disequilibrium rate in EAS populations including the first position, thus representing a single haploblock therein.

### 1000 Genomes assessment on the 15-fold FTO locus

**Fig. 3:**
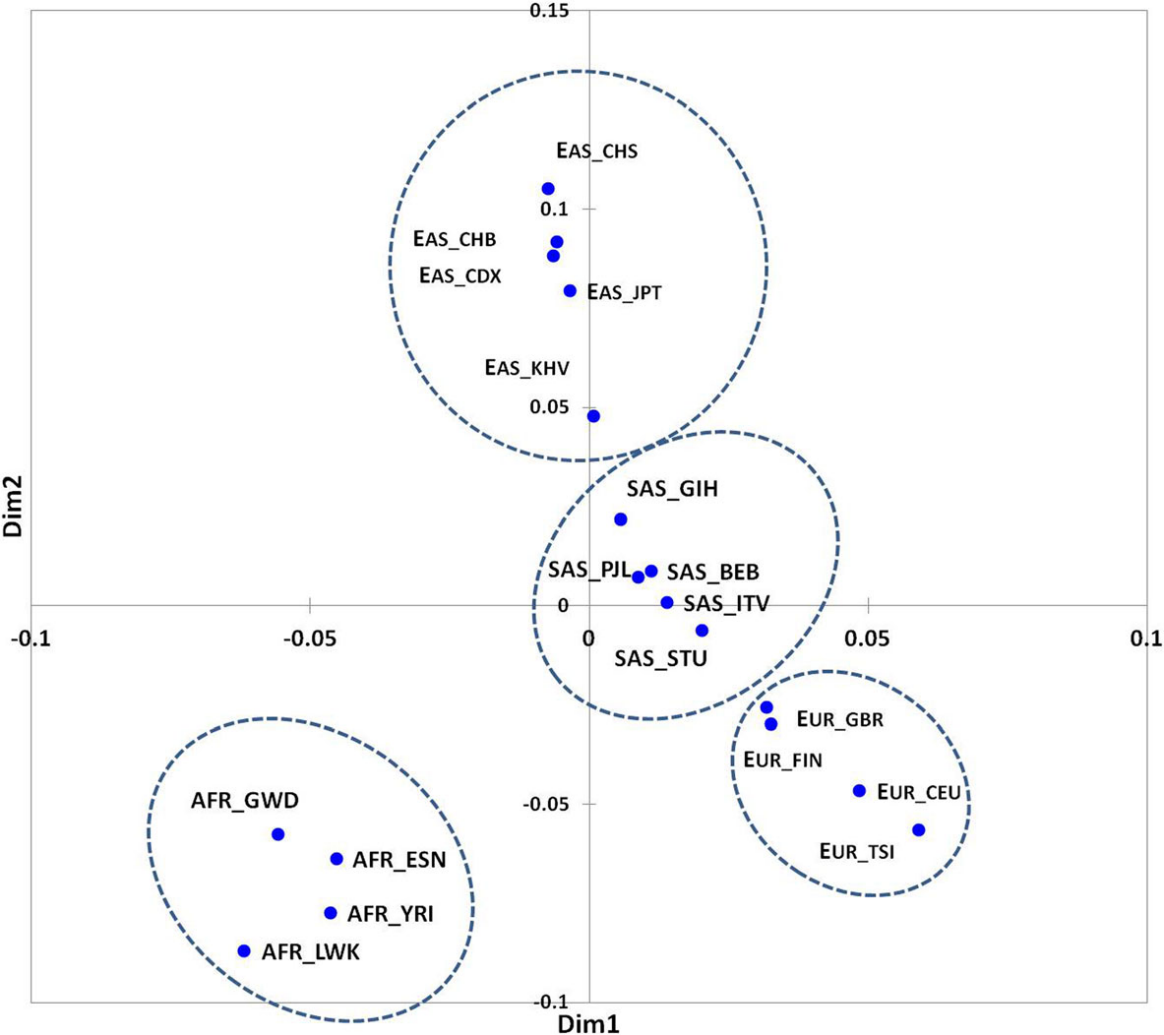
MDS plot based on *Fcon* values of 18 populations based across *FTO* intron 1 15 loci, retrieved from 1000G 4 supergroups (AFR, EAS, SAS, EUR; encircled)

To control for the haplotype consistency, we performed pairwise comparison of haplotype distributions in populations using a conventional *F* statistic ([24]; see methods) analogous to *Fst* ratio, with a subsequent Multi Dimensional Scaling plot creation (XLStat, Inc.; xlstat.com) presented in Fig. 3. We may see that the populations unambiguously cluster into corresponding continental groups, underscoring the consistency of the haplotype clustering.

### Converging to 10 letters haplotype

Based on the Arlequin Amova variance analysis (Table 2; [23, 24]), we excluded positions 2, 5, 7–9 due to their small variations (< 7.1%; Va < 0.011) among groups (Table 2, bold typed), also underlined on haplotype profiles of AFR (Fig. 3) and other populations (not shown). The aim of conversion was to reduce the haplotype variability to core haplotypes suitable to infer the transparent, essential features of population specific haplotype distribution by Principal Component Analysis (PCA) and phylogeny tree plotting.

**Table 2:** Amova (Arlequin 3.5) variation analysis among supergroups (EAS, SAS, EUR, AFR)

Locus	SSD	d.f.	Va	% variation
1	116.99	3	0.04	16.56
2	31.31	3	0.01	**4.81**
3	73.86	3	0.03	14.42
4	74.28	3	0.03	14.53
5	37.86	3	0.01	**5.80**
6	65.43	3	0.02	13.09
7	33.74	3	0.01	**5.69**
8	37.79	3	0.01	**6.30**
9	42.97	3	0.02	**7.09**
10	57.99	3	0.02	9.28
11	57.99	3	0.02	9.28
12	58.13	3	0.02	9.31
13	58.13	3	0.02	9.31
14	62.22	3	0.02	12.45
15	70.26	3	0.03	13.66

*SSD* sum of squared deviations, *Va* variation among groups [24]

Rows with variation percentage less that 8% (subject to removal) are marked with bold in the last column

We thus removed the positions with minimal (4–8%) variation among groups of populations (SAS, EAS, AFR, EUR), which reduced the representation of abundant haploblocks 2 and 3 (Fig. 2) without losing the population specificity of the haplotypes. After the compressing the 15-letter haplotypes to 10-fold ones we observed 6 core alleles, presented in Table 3.

**Table 3:** Frequencies of six 10-fold haplotypes in 4 populations with total frequency > 0.1

	SAS	AFR	EAS	EUR	SUM
GTTAATAACG	0.344	0.195	**0.643**	0.271	1.45
ATTAATAACG	0.267	**0.303**	0.13	0.208	0.91
ACAGGAGGTA	0.118	0.00358	0.0537	**0.297**	0.47
ATTAGAGGCG	0	**0.35**	0	0	0.35
GCAGGAGGTA	0.12	0.0358	0.0785	0.0543	0.29
GTTAGAGGCG	0	**0.101**	0.00199	0	0.10
SUM	**0.85**	**0.99**	**0.91**	**0.83**	

Haplotypes are ranged from the highest population wise frequency (SUM column) down to the lowest

Major population specific haplotype frequences are marked with bold

### FTO intron 1 haplotype evolution

**Fig. 4:**
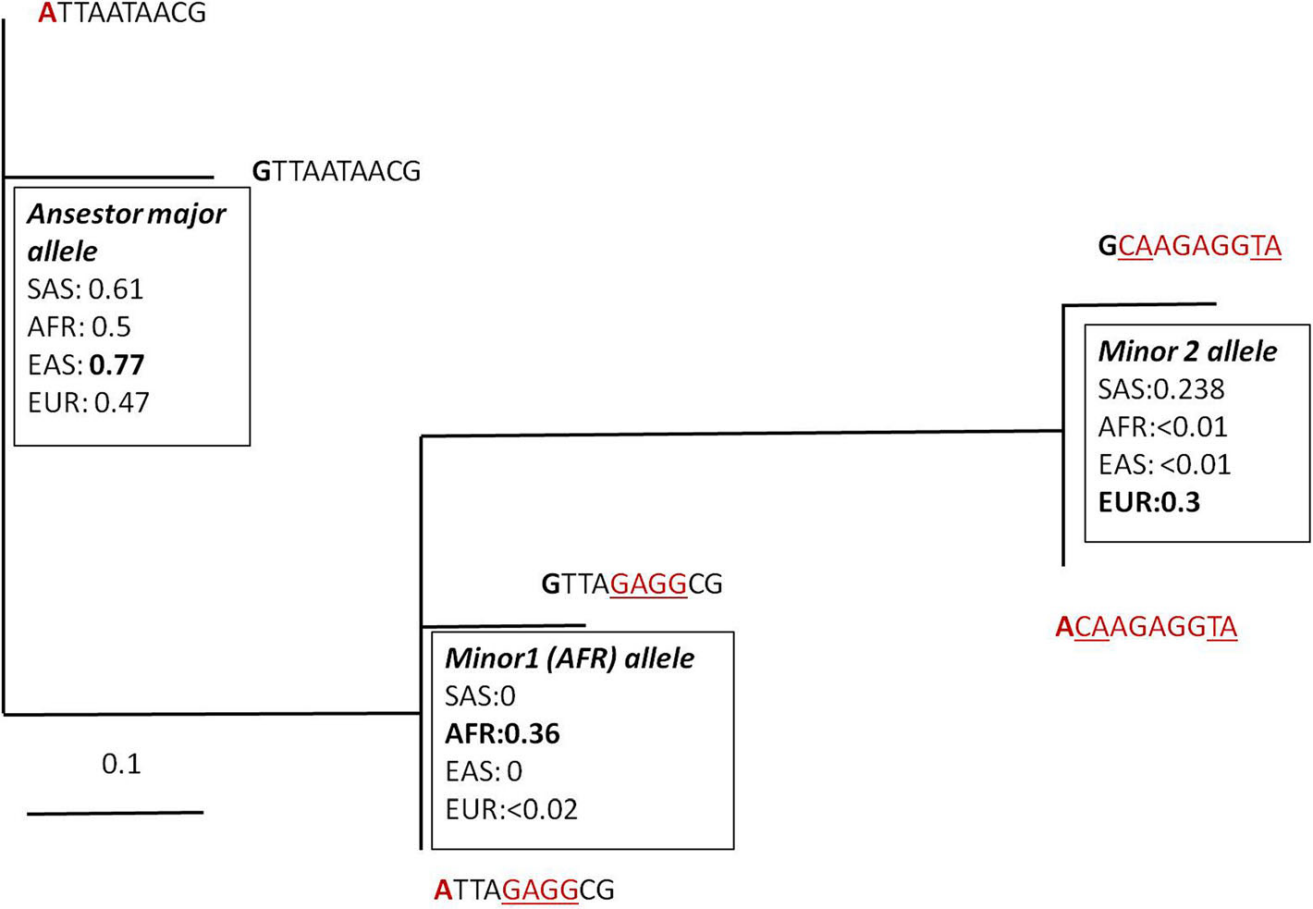
ML haplotypes evolution phylogenetic tree depicting ancestral state and allele frequencies distribution. Compressed 10-letters haplotypes exclude 2, 5, 7–9 positions according to small variation score between populations (Amova results: Table 2; Fig. 2). Frequencies comprise both first position alleles. The risk-associated alleles are colored by red. SNPs diverged from ancestral state are underlined. Bold type corresponds to first position altered in each of allele. The second position is the highest replicated allele rs1421085 (Table 1) affecting adipocyte metabolism (Claussnitzer et al., 2015)

We reconstructed the phylogeny of haplotypes from Table 3 using the DNAML routine from the *phylip* package (Fig. 4). We also excluded the distant first SNP impact in frequencies assessment for transparency. From the locus phylogeny it follows that: a) the major haplotype (population-wide) was present in the ancestral (African) population and comprised low-risk SNP alleles (partially except for the first position) (Fig. 4;’ major allele’); and b) The second ancestor allele maintains haploblock 3 (Fig. 2) inversed (Fig. 4; ‘minor1 allele’), which contains 4 obesity risk substitutions. Further evolution resulted in fixing of European/South Asian specific alleles (Fig. 4, ‘minor2 allele’) consisting totally of risk-associated SNPs variants.

Notably, the major low-risk allele frequency is elevated in EAS/SAS population relative to AFR state and is decreased in EUR populations (Fig. 3; ‘major allele’).

### PCA analysis of haplotype distribution

**Fig. 5:**
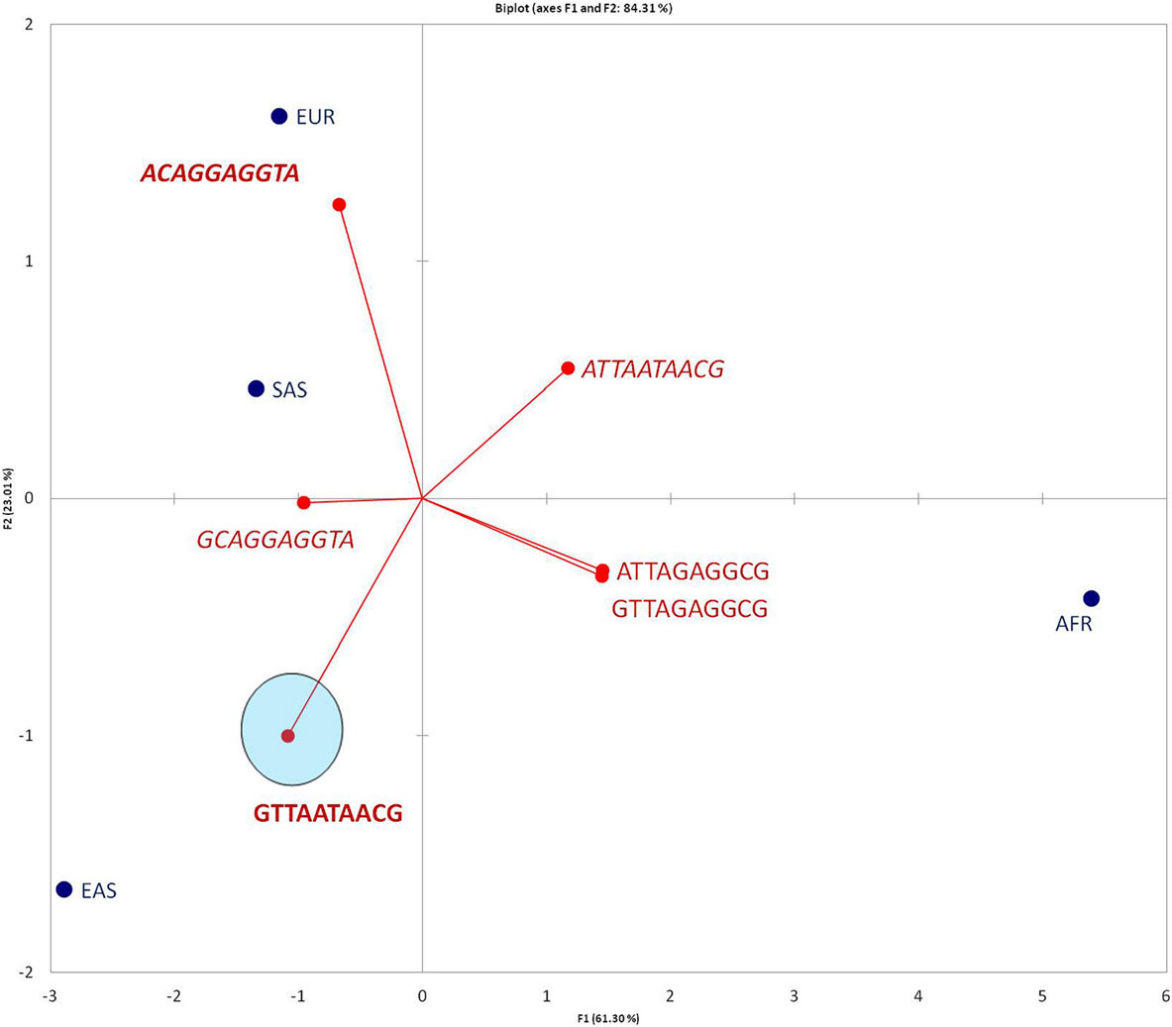
PCA analysis of haplotypes distribution. Population wise major low-risk allele is encircled and bold typed. Risk-associated allele is bold italic and features EUR populations. Two ‘intermediate’ alleles are marked with italic

Based on Table 3, we performed the PCA analysis, presented in Fig. 5, which indicated a distinct distribution of population–specific haplotypes. In particular, population wise Europeans possess the highest ratio of risk-associated alleles (0.3; Fig. 5; Table 3), while EAS populations have the highest low-risk allele ratio (0.64; Table 3). The intermediate allele ATTAATAACG located opposite the EAS specific allele implies that the first position low-risk allele is interlinked (by *r*^*2*^ disequilibrium rate) with the rest of SNPs specifically in EAS populations, contrary to others (Fig. 2). Another intermediate allele located opposite to African-specific haplotypes implies that no polymorphism is observed in African populations for 2, 3, 6, 9, 10 positions according to the SNP frequency profiles (Fig. 2).

### Six major 15-fold haplotypes

**Fig. 6:**
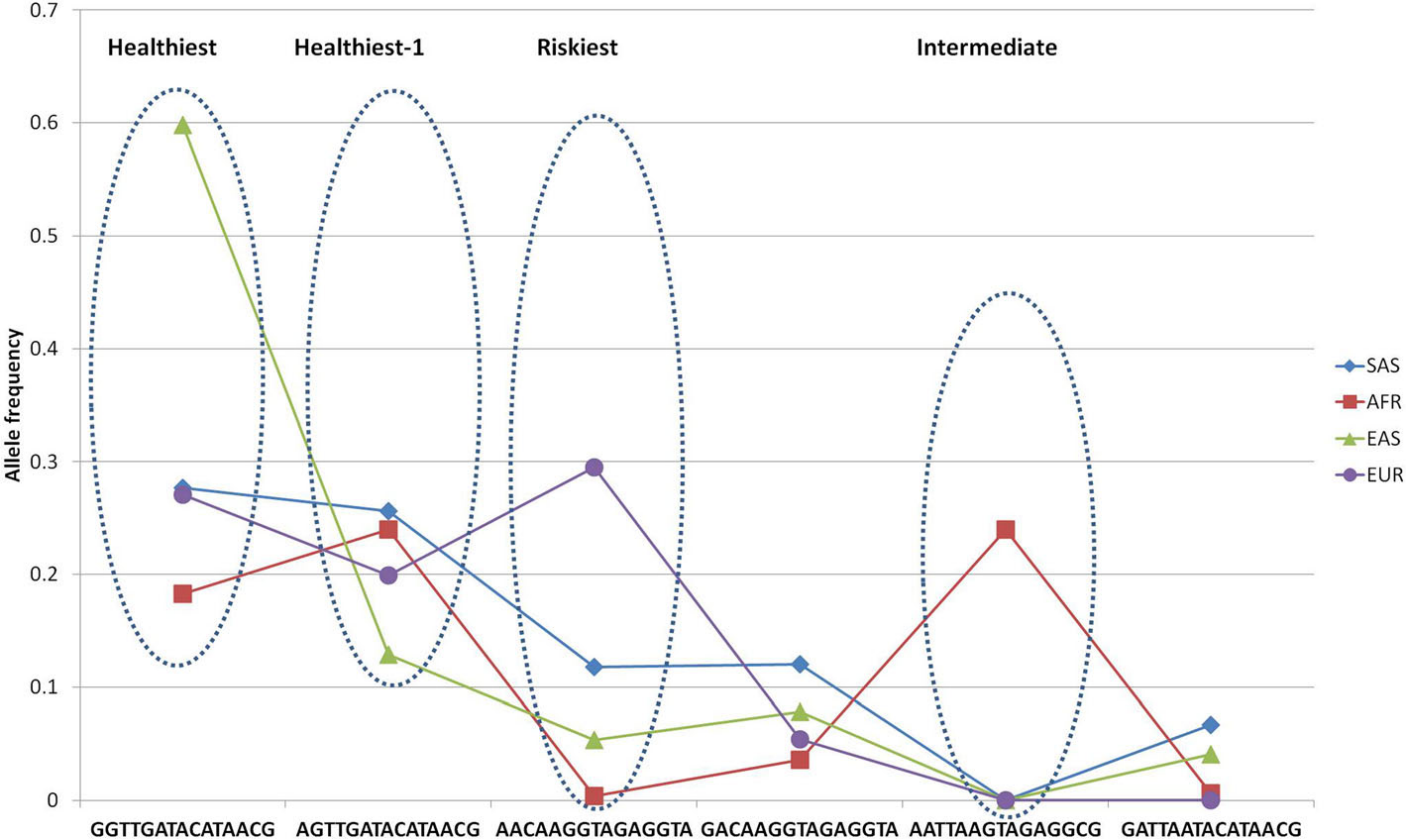
6 major 15-letter haplotypes in 3 populations (Table 4) ‘Healthiest-1’ phenotype maintains altered first position compared to ‘Healthiest’. ‘Intermediate’ comprises more than 1 risk-associated positions (see Fig. 5)

Six target full-sized 15-fold haplotypes corresponding to ones in Table 3 are presented in Table 4 and are plotted in Fig. 6. Two haplotypes (Table 4; N1, 2) correspond to healthy state haplotype except for the first position, which is highly linked in EAS populations only. The highest-risk haplotype (Table 4; N3) is essentially presented in EUR and SAS populations.

**Table 4:** Frequency distribution of six 15-fold haplotypes (total sum per haplotype > 0.1)

N	haplotype	SAS	AFR	EAS	EUR	SUM
1	**GGTTGATACATAACG**	0.277	0.183	**0.598**	0.271	1.329
2	AGTTGATACATAACG	0.256	0.240	0.129	0.199	0.824
3	***AACAAGGTAGAGGTA***	0.118	0.004	0.054	**0.295**	0.470
4	GACAAGGTAGAGGTA	0.120	0.036	0.079	0.054	0.289
5	AATTAAGTAGAGGCG	0.000	0.240	0.000	0.000	0.240
6	GATTAATACATAACG	0.067	0.006	0.041	0.000	0.113
	**SUM**	**0.838**	**0.708**	**0.900**	**0.819**	

The healthiest haplotype is marked in bold. The riskiest haplotype is marked in bold italic. The intermediate AFR-specific haplotype is underlined (see Fig. 5 for details). Haplotypes are ranged from the highest population wise frequency (SUM column) down to the lowest

## Discussion

It was corresponded recently that the *FTO* gene in human has experienced natural selection pressure in the course of evolution [25], possibly featuring population adaptation to the climatic environment, from the hot climate areas to the cold sustaining regions [26]. A range of studies underlined its involvement in thermogenesis [10] and cell metabolism [27] and it was reported that *FTO* affects the obesity trait in age- dependent manner [9, 12]. GWAS reports confirm the association with the trait in all continental supergroups (Additional File 1, Table S1).

At the same time, the 1000G community reports quite distinct ethnospecific haplotype distribution for a vast range of loci [22]. Indeed, use of haplotype analysis revealed drastic differences in allele frequencies across four continental supergroups at *FTO* intron 1 locus (Figs.5, 6). Notably, the distribution of GWAS associated haplotypes often features EAS populations specifically manifesting a highly unimodal major haplotype phenomenon for a range of loci (*FTO*, Fig. 6; *APOE,* [28]), resulting in unequal allele frequencies that are often shifted towards an advantageous one (*FTO*, Fig. 6; *APOE,* [28]).

Haplotype analysis confirmed that the target locus of *FTO* intron 1 is highly interlinked: we observed only 6 major haplotypes encompassing more than 80% of haplotype variation across 4 populations (Fig. 3). Also, we inferred that the ancestral haplotypes in African populations are not observed in other populations. In particular, African haplotypes maintain positions 3, 4, 6, 14, 15 non-variable (Fig. 2), which implies a rapid locus evolution.

The European supergroup maintains a high linkage disequilibrium rate across the GWAS SNPs considered (except for the 1st) and manifests 2 complementary alleles of ‘Ying-Yang’ type [29]. These 14-letter haplotypes encompass 3 top haplotypes in Table 4 (merge of N1, 2 represents the major low-risk allele, and N3 corresponds to minor risk allele) comprising 80% of locus variation for the EUR supergroup.

### Allele dosage

Based on the previous reports [10, 12], the risk-associated allele manifests the trait in a co-dominant manner, so we assessed the inferred genotype distributions at the target locus for European population.

The available data on the Polish population conveys some auxiliary assessments on *FTO* intron 1 locus [30]. In particular, the frequencies of two ‘Yin-Yang’ 4-letter haplotypes (rs1421085, rs1121980, rs9930506, rs9939609) in the Polish population are 53% vs 42% (95% of total haplotype variation), corresponding to low-risk and risk-associated haplotypes [30]. In our EUR cohort we maintain corresponding values of 44% vs 41% ‘Yin-Yang’ alleles for 14 letter haplotypes. Assigning the ‘H’ for low-risk and ‘R’ for risk alleles the inferred Hardy-Weinberg compliant genotypes distribution is: HH: 231(28%), 2RH: 370 (44.8%), RR: 149 (18%) [30]. The ratio of European inferred genotype distribution based on extended haplotypes data is: 153(27%), 296 (50%), 133 (23%). This essentially implies that approximately 20% of the Polish/European population is homozygous for the risk allele and are thus subject to a strong obesity genetic predisposition. The 45–50% heterozygous subjects maintain risk as well, while only around 28% of population doesn’t have any of the obesity risk SNP alleles in *FTO* intron 1 genotype. The ratio for the 4-fold haplotype used in Polish population analysis essentially holds true for the extended 14-letter haplotype (first position is linked in Asian population only; Fig. 5). Thus, the highly unequal haplotype distribution population wise suggests that the risk allele is a highly predisposing genetic factor for obesity specifically in European population. The African population maintains the risk allele restricted to a much smaller risk haplotype size (Figs. 4, 6), while the Asian population maintains an extremely low (less than 0.055) frequency of the highest-risk haplotype (Table 4; Fig. 6), which is abundant in Europeans (0.3).

Notably, while there were no associations of obesity in Polish population with any of the haplotypes observed [30], the age group analyzed in that study was 20 to 40 years old, which presumably means that this locus, long shown by at least 24 GWAS projects (Additional File 1, Table S1) to be associated with genetic predisposition to obesity, has late onset penetrance. Indeed, the recent work on Polish population *FTO* haplotype analysis confirmed the obesity association with *FTO* intron 1 locus [31], but statistical significance held in males only. We observed slightly increased risk haplotype frequency in males (0.34) vs females (0.26) in European populations.

*FTO* implication in disease etiology, besides the reported superenhancer-mediated one in adipocytes, may confer many other mechanistic causes, since intron 1 is highly enriched in densely located transcription factor binding sites (TFBS), some of which overlapping the risk SNPs considered (Table 5). TFBS density elevations were particularly prominent at the 5′ UTR and GWAS SNPs cluster considered, according to ENCODE 161 TF ChIP-seq profiles (*Txn_factor_ChIP* track in UCSC browser (genome.ucsc.edu)). It could also be linked to age related methylation pattern alteration [18–20] impacted by CpG alteration content as underlined in Table 1, since *FTO* reveals a distinctly methylated profile across the gene body in the majority of ENCODE cell lines.

**Table 5:** 15 GWAS SNPs overlap with ENCODE ChIP-Seq TFBS profiles (161 TFs; http://hgdownload.soe.ucsc.edu/goldenPath/hg19/encodeDCC/wgEncodeRegTfbsClustered/)

rs_id	pos	TF	Score	Peak size, bp
**rs9940128**	**53,800,753**	**EP300**	**763**	390
rs9940128	53,800,753	FOSL2	424	260
rs9940128	53,800,753	NFIC	351	544
rs9940128	53,800,753	SP1	273	336
rs9940128	53,800,753	JUND	211	332
rs9940128	53,800,753	TEAD4	173	440
rs9940128	53,800,753	FOXA2	183	290
rs9940128	53,800,753	MYBL2	403	616
rs9940128	53,800,753	FOS	339	283
rs9940128	53,800,753	STAT3	241	336
rs1421085	53,800,953	FOS	339	283
rs1121980	53,809,246	USF1	144	264
rs1121980	53,809,246	SPI1	216	263
rs62033400	53,811,787	MAFK	266	287
rs8050136	53,816,274	EP300	268	330
rs8050136	53,816,274	FOXA1	204	240
rs8050136	53,816,274	GATA3	231	296
rs7202116	53,821,614	FOS	299	275
**rs7202116**	**53,821,614**	**MAFK**	**1000**	290
**rs7202116**	**53,821,614**	**MAFF**	**852**	280
rs17817964	53,828,065	GATA3	213	296

Score varies from 1 to 1000

Highly scored TFs are marked with bold

## Conclusions

The ultimate goal of the work was to elucidate and underscore rapid population-specific fixation of the obesity associated genetic variant (haplotype) in European populations based on *FTO* intron 1 expanded haplotype frequency profiling in the major continental supergroups of the1000 Genome Project. We also observed quite rapid evolutionary rate of the locus displayed by European populations, implying that a selective sweep might have taken place in its evolutionary course [25]. Our conclusions corroborate the BMI WHO report summed up in [21], which contrasted obesity risks in East Asian vs European populations.

## Additional file

Additional file 1: Table S1. List of GWAS publications for the 15 SNPs considered. Table S2. Description of 1000G populations employed. (XLSX 37 kb)

## Abbreviations

AMOVA: Analysis of Molecular Variance
BMI: Body Mass Index
ENCODE: Encyclopedia of DNA Elements
FTO: Fat mass and obesity-associated gene
GWAS: Genome Wide Association Studies
PCA: Principal Components Analysis
TFBS: transcription factor binding site
UCNE: ultra conserved non-coding elements
